# Supplementary material for: Mlf mediates proteotoxic response via formation of cellular foci for protein folding and degradation in Giardia
Source: PLoS Pathog. 2024 Oct 21;20(10):e1012617. doi: 10.1371/journal.ppat.1012617 (PMC11527388; doi:10.1371/journal.ppat.1012617)
Supplement: S1 Fig — (A) Full phylogenetic tree of GiMlf’s homologs from Fig 1 supplied with support values and protein identifiers. (B) Multiple sequence alignment of GiMlf and its homologs from different eukaryotic supergroups with indicated secondary structure of GiMlf (α—alpha helix (blue), β—beta strand (yellow), η - 3–10 helix, T–turn). The highlighted positions within the sequence show conservation in at least 70% of the sequences. (PDF) [file ppat.1012617.s001.pdf]

A

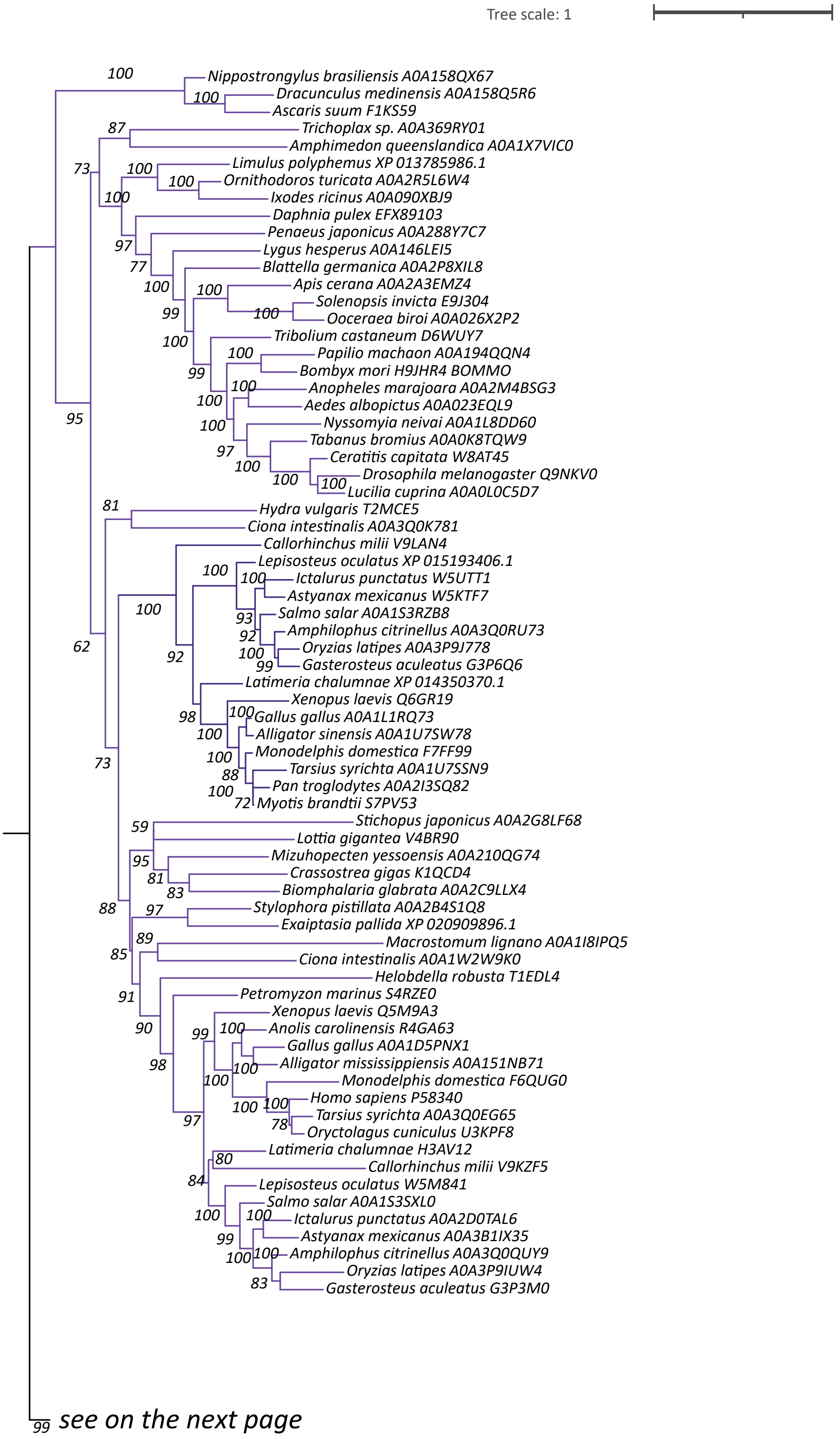
Tree scale: 1
Nippostrongylus brasiliensis A0A158QX67
Dracunculus medinensis A0A158Q5R6
Ascaris suum F1KS59
Trichoplax sp. A0A369RY01
Amphimedon queenslandica A0A1X7VIC0
Limulus polyphemus XP 013785986.1
Ornithodoros turicata A0A2R5L6W4
Ixodes ricinus A0A090XBJ9
Daphnia pulex EFX89103
Penaeus japonicus A0A288Y7C7
Lygus hesperus A0A146LEI5
Blattella germanica A0A2P8XIL8
Apis cerana A0A2A3EMZ4
Solenopsis invicta E9J304
Ooceraea biroi A0A026X2P2
Tribolium castaneum D6WUY7
Papilio machaon A0A194QQN4
Bombyx mori H9JHR4 BOMMO
Anopheles marajoara A0A2M4BSG3
Aedes albopictus A0A023EQL9
Nyssomyia neivai A0A1L8DD60
Tabanus bromius A0A0K8TQW9
Ceratitis capitata W8AT45
Drosophila melanogaster Q9NKV0
Lucilia cuprina A0A0L0C5D7
Hydra vulgaris T2MCE5
Ciona intestinalis A0A3Q0K781
Callorhinchus milii V9LAN4
Lepisosteus oculatus XP 015193406.1
Ictalurus punctatus W5UTT1
Astyanax mexicanus W5KTF7
Salmo salar A0A1S3RZB8
Amphilophus citrinellus A0A3Q0RU73
Oryzias latipes A0A3P9J778
Gasterosteus aculeatus G3P6Q6
Latimeria chalumnae XP 014350370.1
Xenopus laevis Q6GR19
Gallus gallus A0A1L1RQ73
Alligator sinensis A0A1U7SW78
Monodelphis domestica F7FF99
Tarsius syrichta A0A1U7SSN9
Pan troglodytes A0A2I3SQ82
Myotis brandtii S7PV53
Stichopus japonicus A0A2G8LF68
Lottia gigantea V4BR90
Mizuhopecten yessoensis A0A210QG74
Crassostrea gigas K1QCD4
Biomphalaria glabrata A0A2C9LLX4
Stylophora pistillata A0A2B4S1Q8
Exaiptasia pallida XP 020909896.1
Macrostomum lignano A0A1I8IPQ5
Ciona intestinalis A0A1W2W9K0
Helobdella robusta T1EDL4
Petromyzon marinus S4RZE0
Xenopus laevis Q5M9A3
Anolis carolinensis R4GA63
Gallus gallus A0A1D5PNX1
Alligator mississippiensis A0A151NB71
Monodelphis domestica F6QUG0
Homo sapiens P58340
Tarsius syrichta A0A3Q0EG65
Oryctolagus cuniculus U3KPF8
Latimeria chalumnae H3AV12
Callorhinchus milii V9KZF5
Lepisosteus oculatus W5M841
Salmo salar A0A1S3SXL0
Ictalurus punctatus A0A2D0TAL6
Astyanax mexicanus A0A3B1IX35
Amphilophus citrinellus A0A3Q0QUY9
Oryzias latipes A0A3P9IUW4
Gasterosteus aculeatus G3P3M0
99 see on the next page

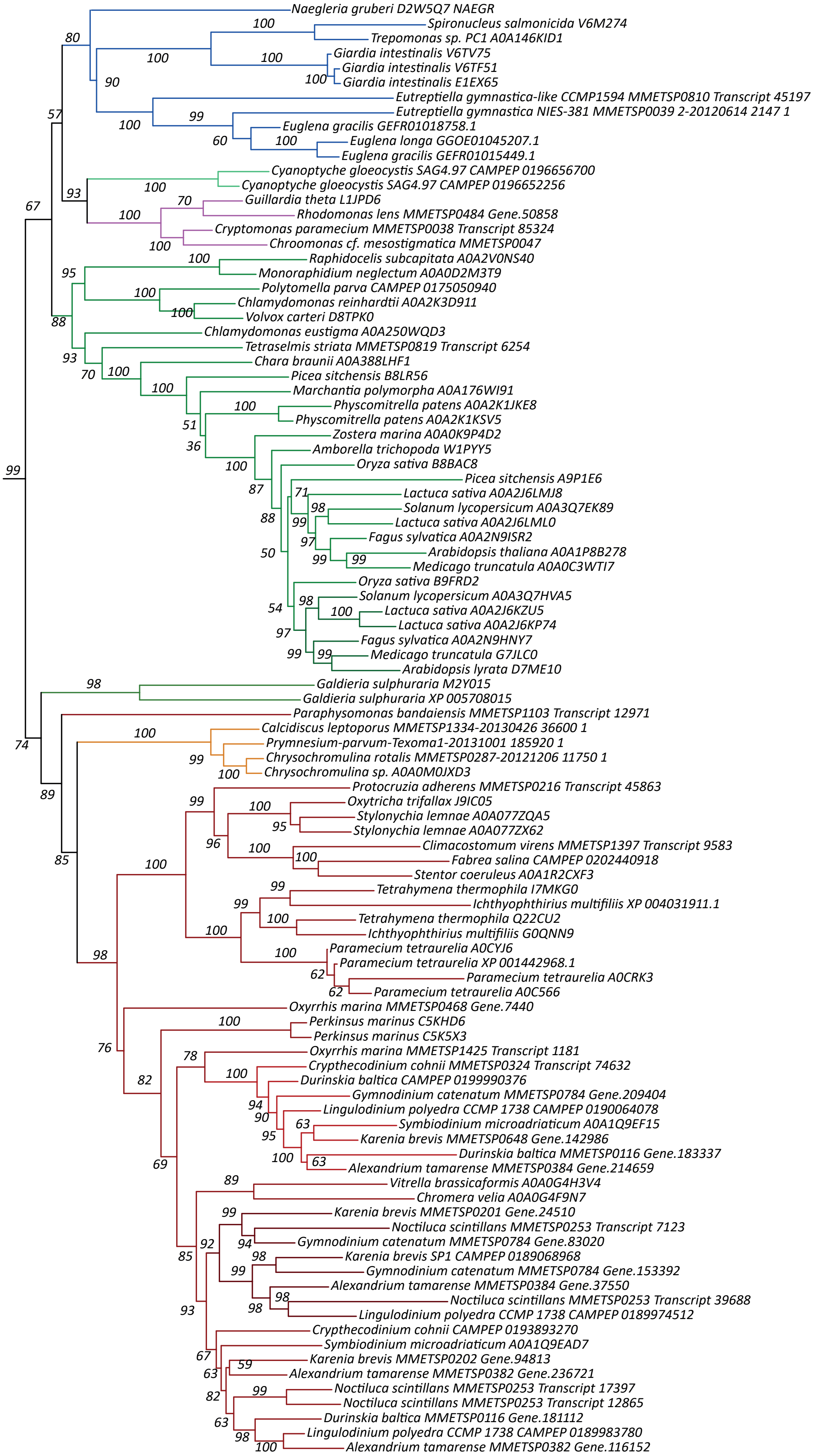
Naegleria gruberi D2W5Q7 NAEGR
Spironucleus salmonicida V6M274
Trepomonas sp. PC1 A0A146KID1
Giardia intestinalis V6TV75
Giardia intestinalis V6TF51
Giardia intestinalis E1EX65
Eutreptiella gymnastica-like CCMP1594 MMETSP0810 Transcript 45197
Eutreptiella gymnastica NIES-381 MMETSP0039 2-20120614 2147 1
Euglena gracilis GEFR01018758.1
Euglena longa GGOE01045207.1
Euglena gracilis GEFR01015449.1
Cyanoptyche gloeocystis SAG4.97 CAMPEP 0196656700
Cyanoptyche gloeocystis SAG4.97 CAMPEP 0196652256
Guillardia theta L1JPD6
Rhodomonas lens MMETSP0484 Gene.50858
Cryptomonas paramecium MMETSP0038 Transcript 85324
Chroomonas cf. mesostigmatica MMETSP0047
Raphidocelis subcapitata A0A2V0NS40
Monoraphidium neglectum A0A0D2M3T9
Polytomella parva CAMPEP 0175050940
Chlamydomonas reinhardtii A0A2K3D911
Volvox carteri D8TPK0
Chlamydomonas eustigma A0A250WQD3
Tetraselmis striata MMETSP0819 Transcript 6254
Chara braunii A0A388LHF1
Picea sitchensis B8LR56
Marchantia polymorpha A0A176WI91
Physcomitrella patens A0A2K1JKE8
Physcomitrella patens A0A2K1KSV5
Zostera marina A0A0K9P4D2
Amborella trichopoda W1PYY5
Oryza sativa B8BAC8
Picea sitchensis A9P1E6
Lactuca sativa A0A2J6LMJ8
Solanum lycopersicum A0A3Q7EK89
Lactuca sativa A0A2J6LML0
Fagus sylvatica A0A2N9ISR2
Arabidopsis thaliana A0A1P8B278
Medicago truncatula A0A0C3WTI7
Oryza sativa B9FRD2
Solanum lycopersicum A0A3Q7HVA5
Lactuca sativa A0A2J6KZU5
Lactuca sativa A0A2J6KP74
Fagus sylvatica A0A2N9HNY7
Medicago truncatula G7JLC0
Arabidopsis lyrata D7ME10
Galdieria sulphuraria M2Y015
Galdieria sulphuraria XP 005708015
Paraphysomonas bandaiensis MMETSP1103 Transcript 12971
Calcidiscus leptoporus MMETSP1334-20130426 36600 1
Prymnesium-parvum-Texoma1-20131001 185920 1
Chrysochromulina rotalis MMETSP0287-20121206 11750 1
Chrysochromulina sp. A0A0M0JXD3
Protocruzia adherens MMETSP0216 Transcript 45863
Oxytricha trifallax J9IC05
Stylonychia lemnae A0A077ZQA5
Stylonychia lemnae A0A077ZX62
Climacostomum virens MMETSP1397 Transcript 9583
Fabrea salina CAMPEP 0202440918
Stentor coeruleus A0A1R2CXF3
Tetrahymena thermophila I7MKG0
Ichthyophthirius multifiliis XP 004031911.1
Tetrahymena thermophila Q22CU2
Ichthyophthirius multifiliis G0QNN9
Paramecium tetraurelia A0CYJ6
Paramecium tetraurelia XP 001442968.1
Paramecium tetraurelia A0CRK3
Paramecium tetraurelia A0C566
Oxyrrhis marina MMETSP0468 Gene.7440
Perkinsus marinus C5KHD6
Perkinsus marinus C5K5X3
Oxyrrhis marina MMETSP1425 Transcript 1181
Crypthecodinium cohnii MMETSP0324 Transcript 74632
Durinskia baltica CAMPEP 0199990376
Gymnodinium catenatum MMETSP0784 Gene.209404
Lingulodinium polyedra CCMP 1738 CAMPEP 0190064078
Symbiodinium microadriaticum A0A1Q9EF15
Karenia brevis MMETSP0648 Gene.142986
Durinskia baltica MMETSP0116 Gene.183337
Alexandrium tamarense MMETSP0384 Gene.214659
Vitrella brassicaformis A0A0G4H3V4
Chromera velia A0A0G4F9N7
Karenia brevis MMETSP0201 Gene.24510
Noctiluca scintillans MMETSP0253 Transcript 7123
Gymnodinium catenatum MMETSP0784 Gene.83020
Karenia brevis SP1 CAMPEP 0189068968
Gymnodinium catenatum MMETSP0784 Gene.153392
Alexandrium tamarense MMETSP0384 Gene.37550
Noctiluca scintillans MMETSP0253 Transcript 39688
Lingulodinium polyedra CCMP 1738 CAMPEP 0189974512
Crypthecodinium cohnii CAMPEP 0193893270
Symbiodinium microadriaticum A0A1Q9EAD7
Karenia brevis MMETSP0202 Gene.94813
Alexandrium tamarense MMETSP0382 Gene.236721
Noctiluca scintillans MMETSP0253 Transcript 17397
Noctiluca scintillans MMETSP0253 Transcript 12865
Durinskia baltica MMETSP0116 Gene.181112
Lingulodinium polyedra CCMP 1738 CAMPEP 0189983780
Alexandrium tamarense MMETSP0382 Gene.116152

B

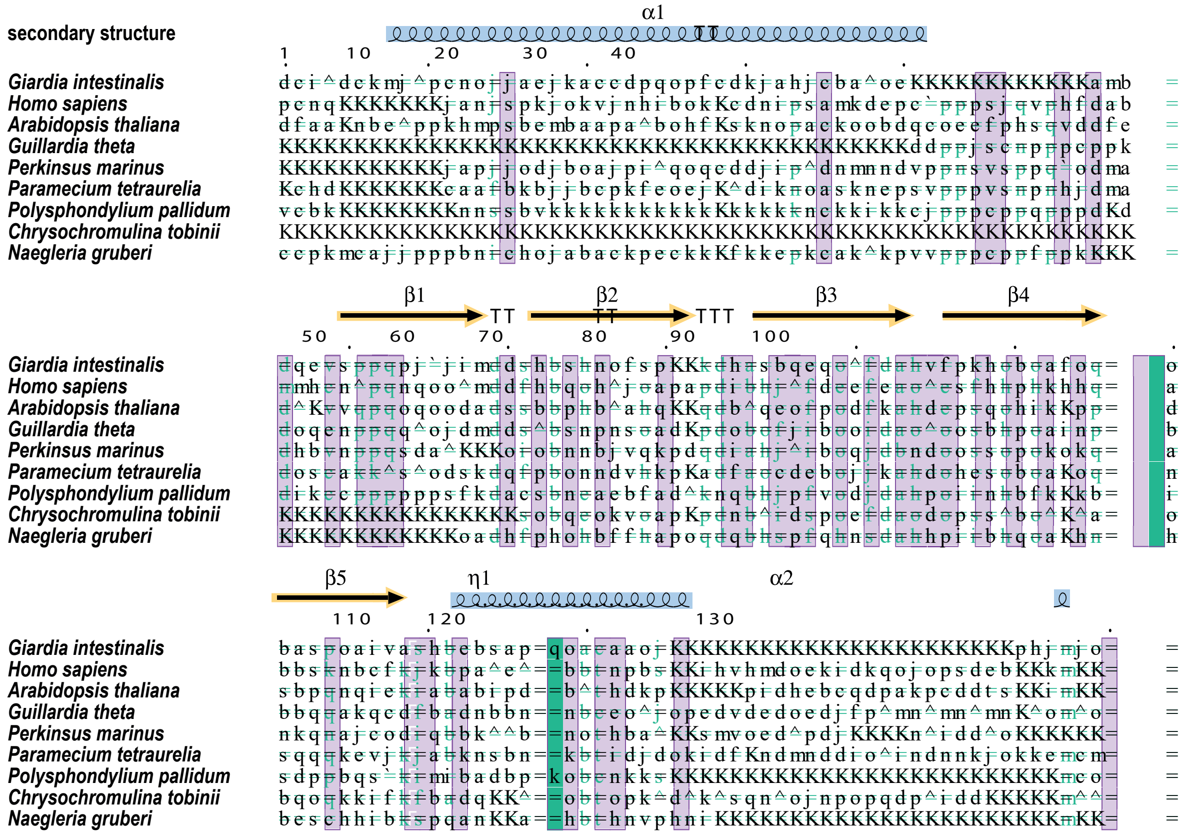
secondary structure
α1
β1
β2
β3
β4
β5
η1
α2
Giardia intestinalis
Homo sapiens
Arabidopsis thaliana
Guillardia theta
Perkinsus marinus
Paramecium tetraurelia
Polysphondylium pallidum
Chrysochromulina tobinii
Naegleria gruberi
