# Supplementary figures and images for: Mlf mediates proteotoxic response via formation of cellular foci for protein folding and degradation in Giardia
Source: PLoS Pathog. 2024 Oct 21;20(10):e1012617. doi: 10.1371/journal.ppat.1012617 (PMC11527388; doi:10.1371/journal.ppat.1012617)

**A**

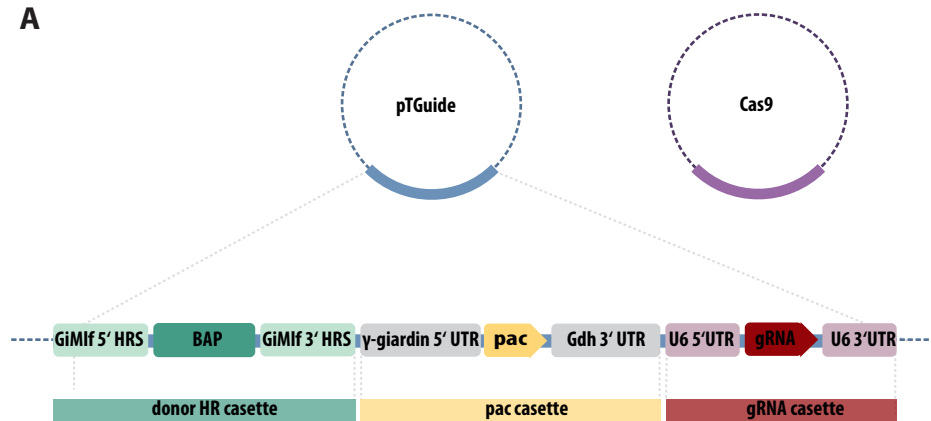

**B**

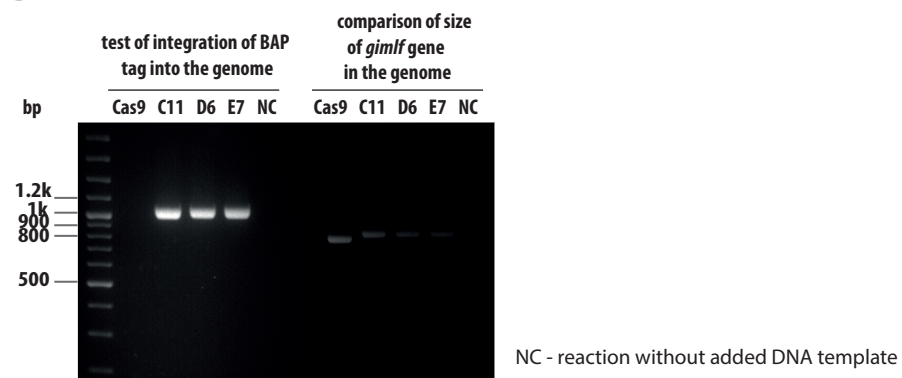

test of integration of BAP tag into the genome

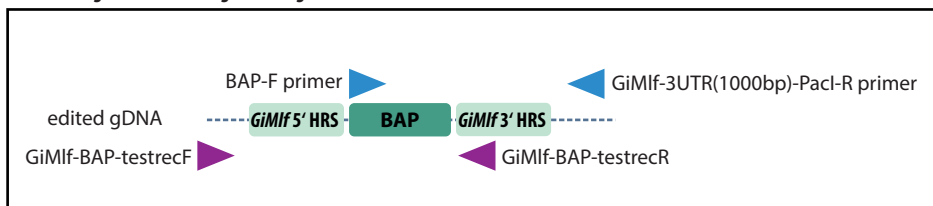

comparison of size of *gimif* in the genome

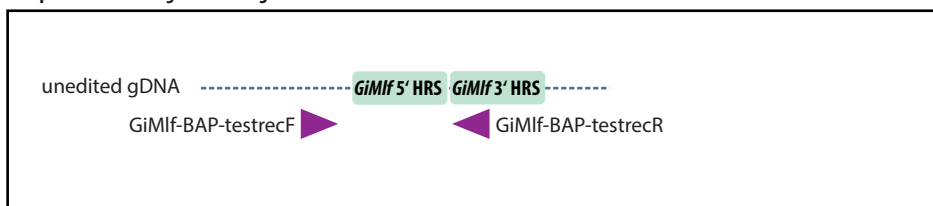

**C**

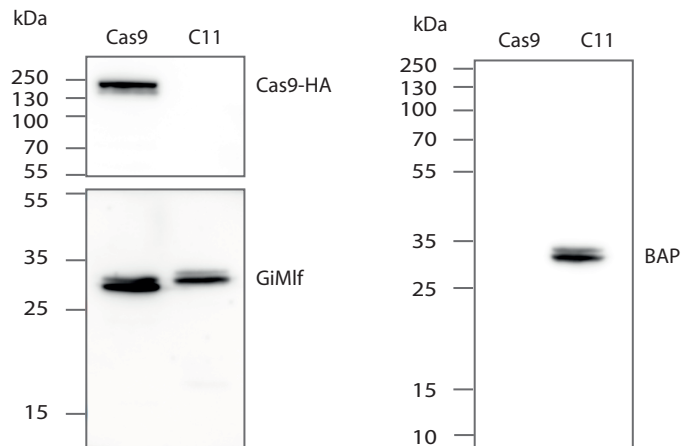

Supplement: S2 Fig — (A) Schematic representation of the constructs used for in situ tagging of GiMlf with the BAP tag using the CRISPR/Cas9 system. In this case, the pac cassette, which provides resistance to puromycin is not part of the repair template. (B) Left—PCR amplification of the integrated BAP tag in the genomes of subcloned populations C11, D6, and E7 of Giardia modified by CRISPR/Cas9. The Cas9 cell line served as the parental cell line, NC-negative control. Right–PCR amplification of the genomic region containing the gimlf gene in modified and control lineages. One of the amplification primers was outside the recombination regions used for the integration of the BAP tag. Only one band was present in all the tested subclones, showing that the BAP tag was integrated into all genomic copies of gimlf in the modified lineages. (C) Western blot of whole cell lysates of the subcloned endogenously BAP-tagged GiMlf cell line (C11) and a control cell line detecting the expression of Cas9-HA (top panel) with anti-HA antibody, the shift in size and the presence of only a single double band when detected with anti-GiMlf polyclonal antibody (middle panel), and the presence of the BAP tag using anti-BAP antibody. (PDF) [file ppat.1012617.s002.pdf]

**A**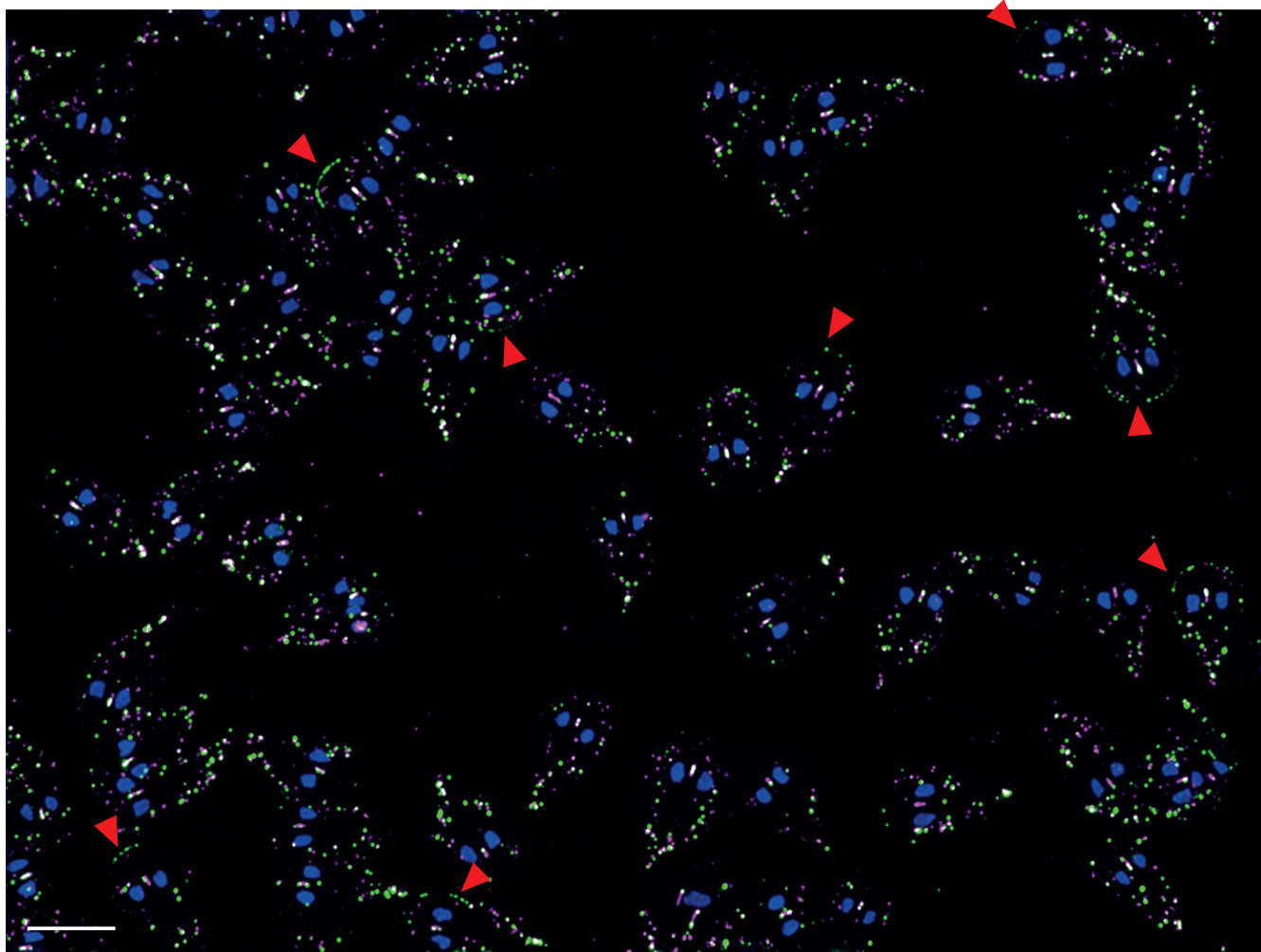**B**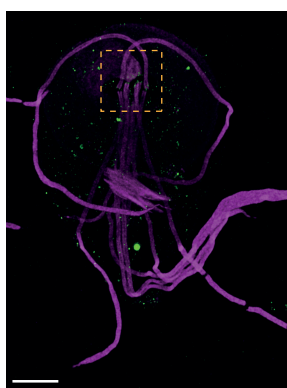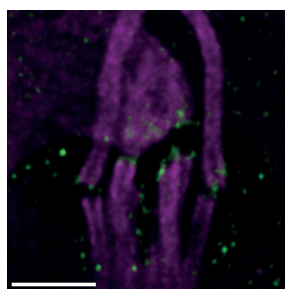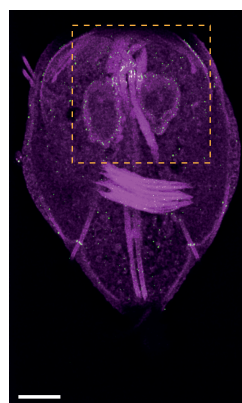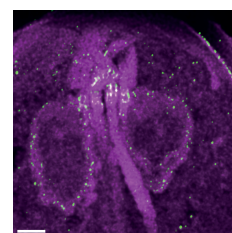

Supplement: S3 Fig — (A) Wide field image of immunofluorescence analysis of GiMlf (green) and mitosomes (magenta), nuclei stained with DAPI (blue), scale bar 10 μm, red arrowheads highlight the presence of GiMlf at disc margin (B) Full images of expansion microscopy images from Fig 2. Scale bars of full images: 10 μm. Scale bars of enlarged sections: 4 μm. (PDF) [file ppat.1012617.s003.pdf]

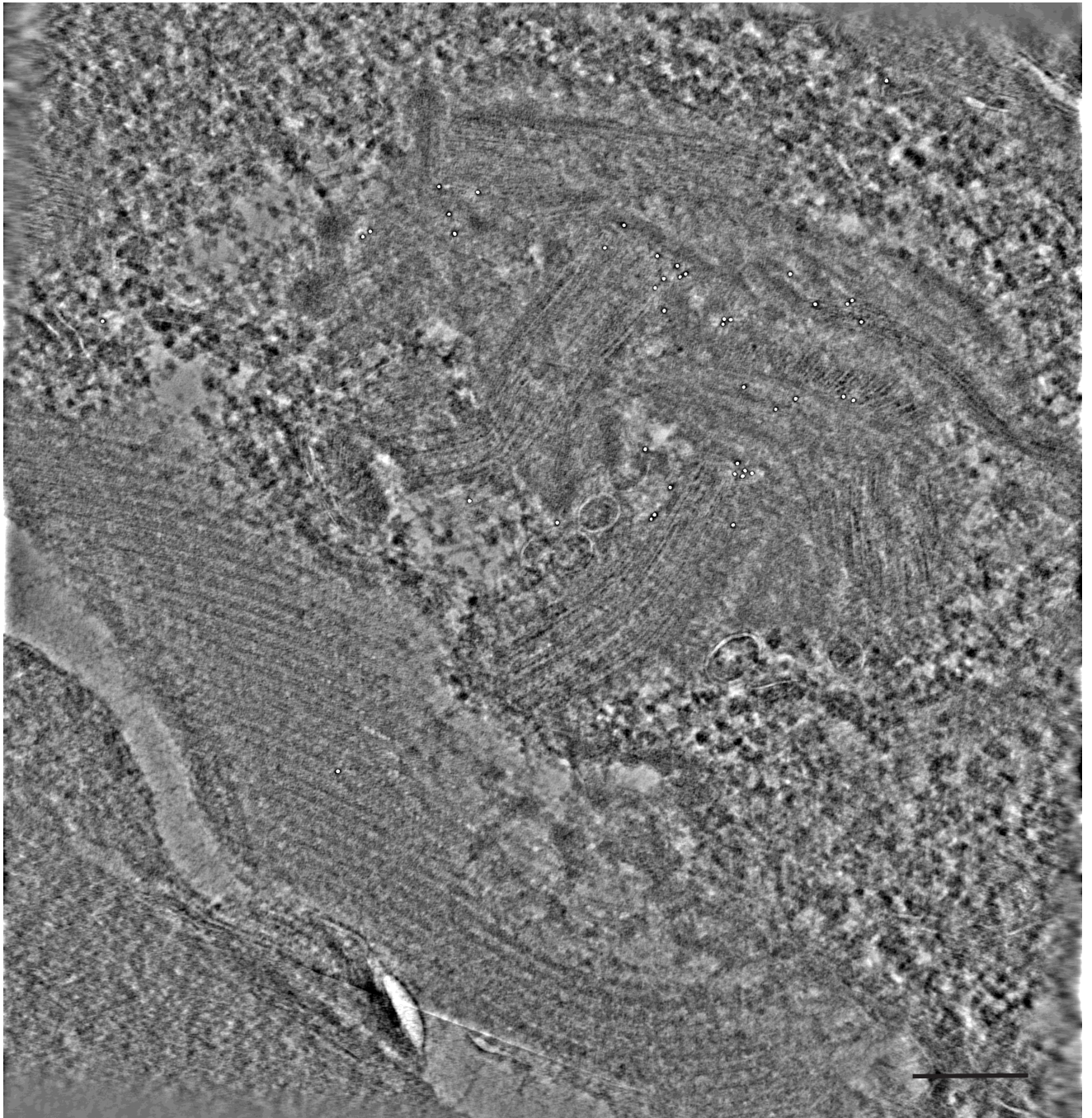

Supplement: S4 Fig — (PDF) [file ppat.1012617.s004.pdf]

**A**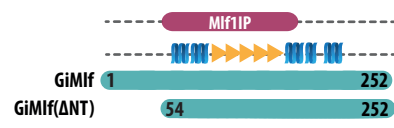**B**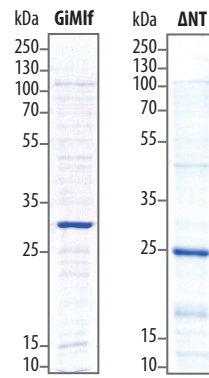**C**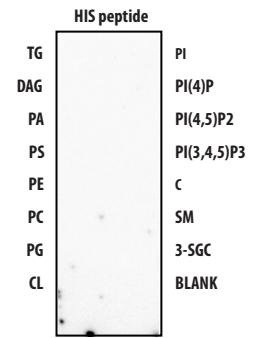

Supplement: S5 Fig — (A) Schematic representation of GiMlf-His protein and its truncated forms with the indicated Mlf1IP domain, secondary structure, and positions of truncation of the individual forms. NT–N-terminal domain, CT–C- terminal domain. (B) SDS-electrophoresis of purified recombinant proteins stained with Coomassie stain. (C). His peptide was used as a negative control for the incubation with the lipid strip, the peptide was detected by an anti-His antibody, TG–triglyceride, DAG–diacylglycerol, PA–phosphatidic acid, PS–phosphatidylserine, PE–phosphatidylethanolamine, PC–phosphatidylcholine, PG–phosphatidylglycerol, CL–cardiolipin, PI–phosphatidylinositol, C–cholesterol, SM–sphingomyelin, 3-SGC– 3-sulfogalactosylceramide, PI(4)P–phosphatidylinositol (4)-phosphate, PI(4,5)P2 –phosphatidylinositol (4,5)-bisphosphate, PI(3,4,5)P3 –phosphatidylinositol (3,4,5)-trisphosphate. (PDF) [file ppat.1012617.s005.pdf]

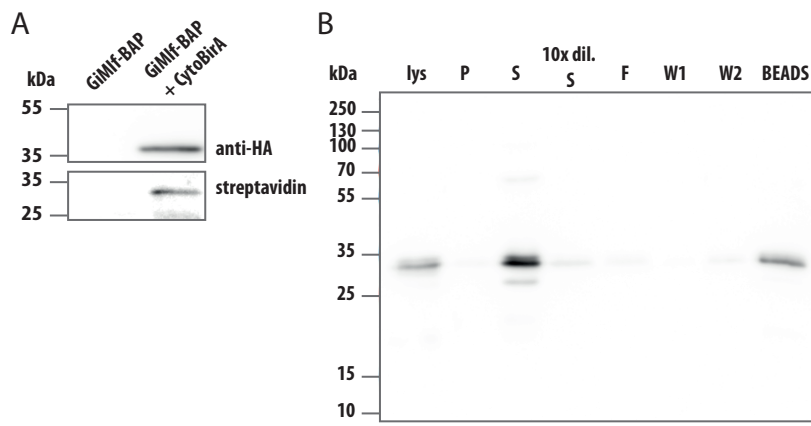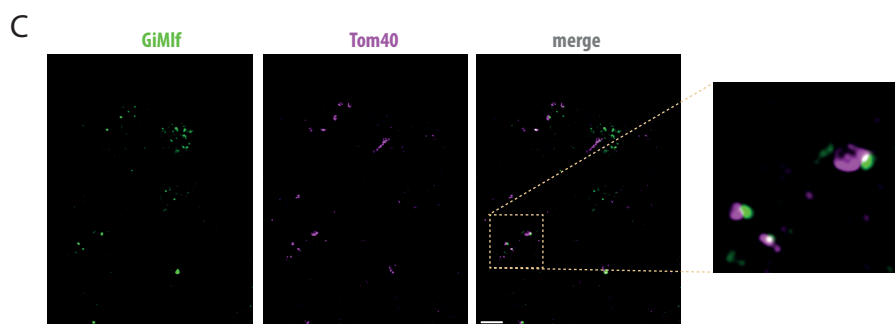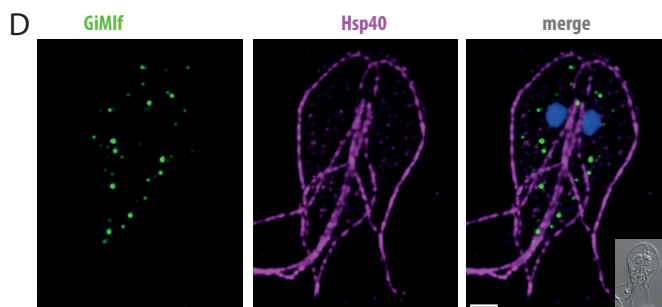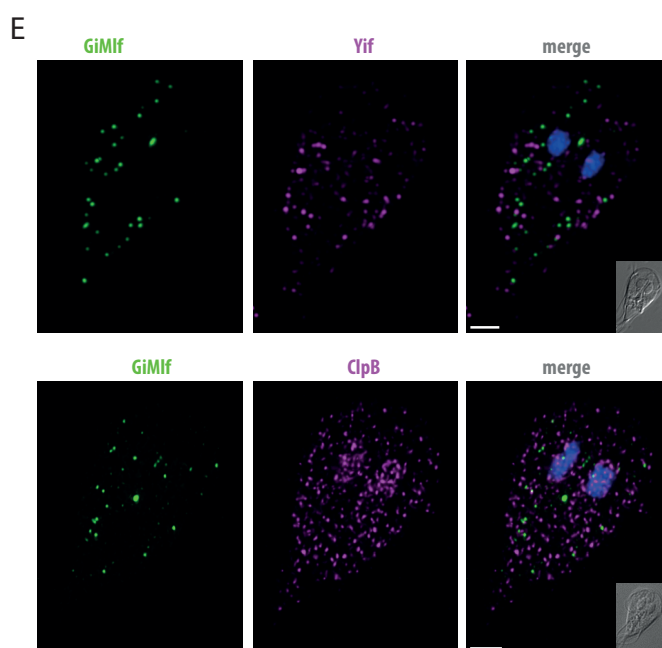

Supplement: S6 Fig — (A) Western blot of the whole cell lysates of the BAP-tagged GiMlf cell line with and without CytoBirA detecting the expression of BirA-HA with anti-HA antibody and the successful biotinylation of BAP-tagged GiMlf using streptavidin. (B) Western blot of the isolation process by biotin affinity purification of BAP-tagged GiMlf and its interaction partners. The ten-fold diluted supernatant of crosslinked proteins was incubated with the streptavidin coated beads. GiMlf-BAP was detected by an anti-BAP antibody. Lys–lysate, P–pellet, S–supernatant, dil–diluted, F–flowthrough, W–wash. (C) Localization of GiMlf and Tom40 in Giardia using expansion microscopy. The cells were stained with anti-BAP antibody (green) and anti-Tom40 antibody (magenta). A single layer is shown. Scale bars: 5 μm. (D) The presence of Hsp40 in the axonemes. The cells were stained with an anti-BAP antibody (green) and an anti-V5 antibody (magenta). Nucleic DNA was stained with DAPI (blue), DIC image of corresponding cell is shown in corner of the merged image. Scale bar: 2 μm. (E) Localization of BAP-tagged GiMlf (anti-BAP antibody, green) and its putative interaction partners isolated in the biotin affinity purification assay (anti-V5, magenta) by confocal fluorescence microscopy. DIC image of corresponding cell is shown in corner of the merged image. Scale bars: 2 μm. (PDF) [file ppat.1012617.s006.pdf]

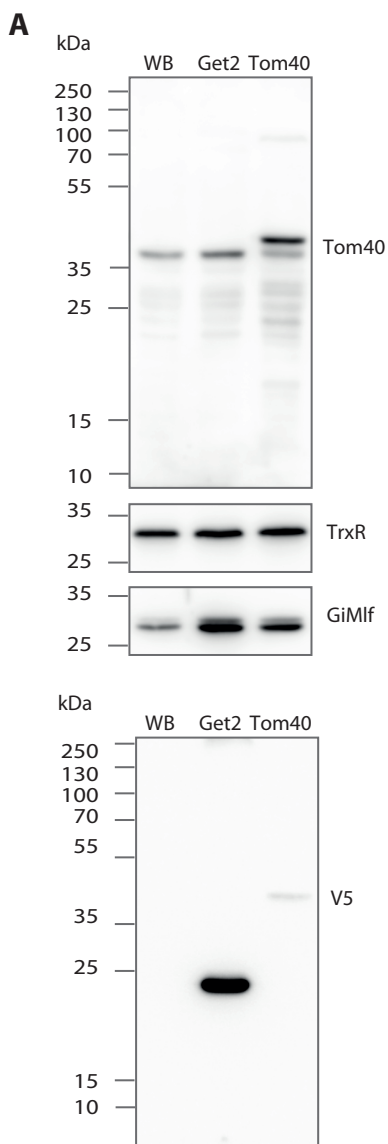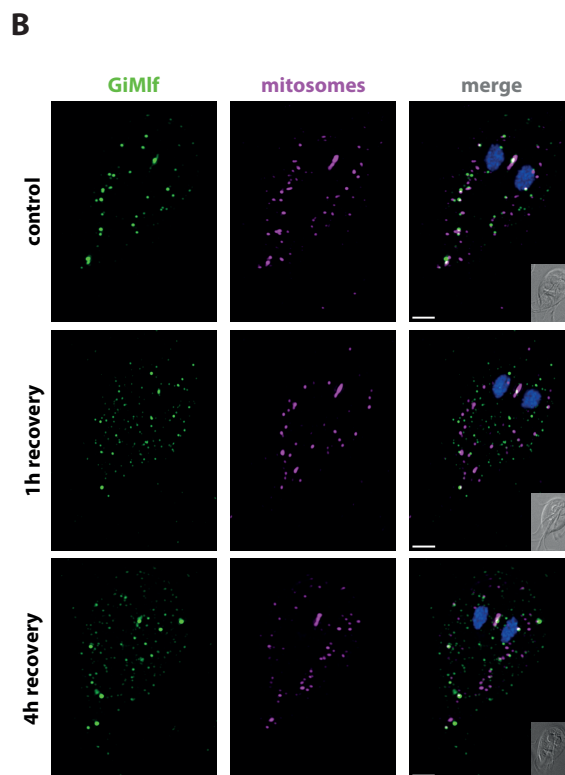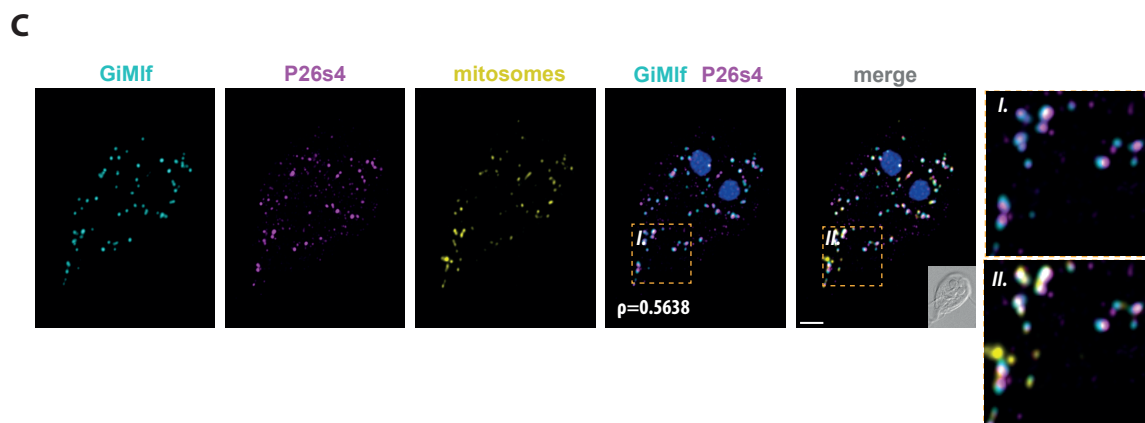

Supplement: S7 Fig — The cells were stained with anti-BAP antibody (cyan), anti-V5 antibody (magenta), and anti-GL50803_9296 antibody (yellow, mitosomal marker). DNA was stained with DAPI (blue). The enlarged images show a single layer of the image stack. Pearson’s correlation coefficient (ρ = 0.5638) was calculated for the subsection of GiMlf and P26s4 that colocalizes in the proximity of mitosomes, DIC images of the corresponding cells are shown in the corner of the merged images. All scale bars: 2 μm. (PDF) [file ppat.1012617.s007.pdf]

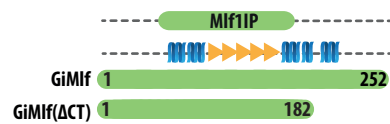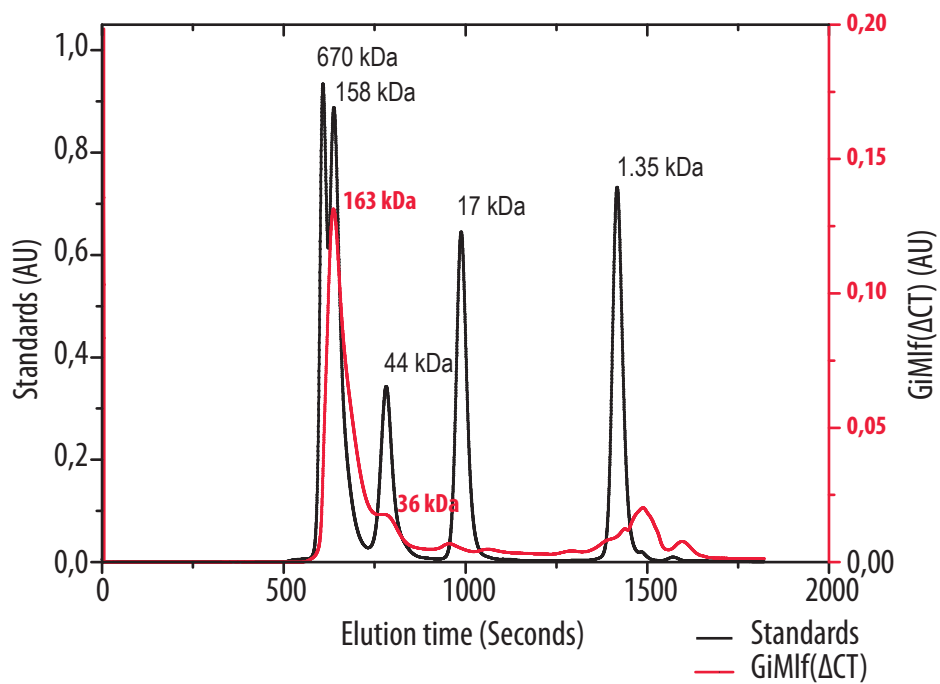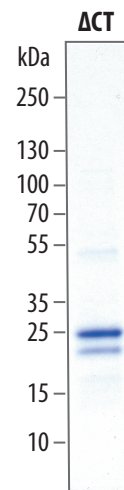

Supplement: S8 Fig — The molecular weight of GiMlf(ΔCT) species was estimated using the logistic model in the CurveExpert software based on the elution volume and the known molecular weight of the standards. (PDF) [file ppat.1012617.s008.pdf]

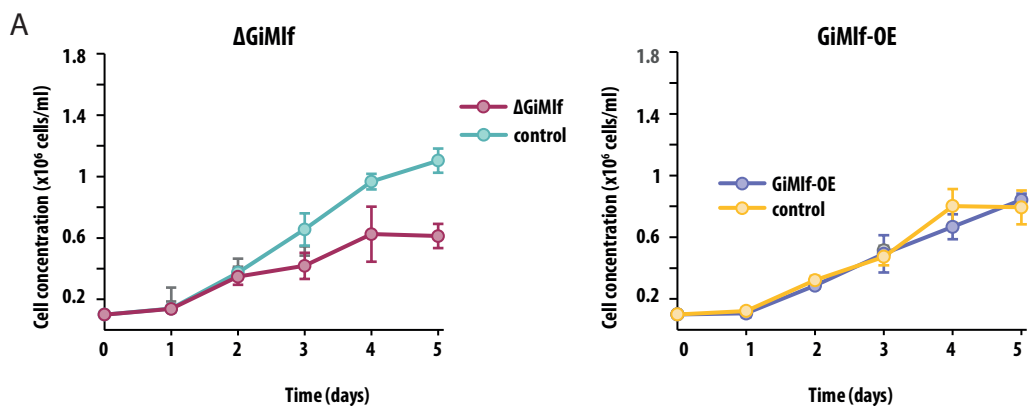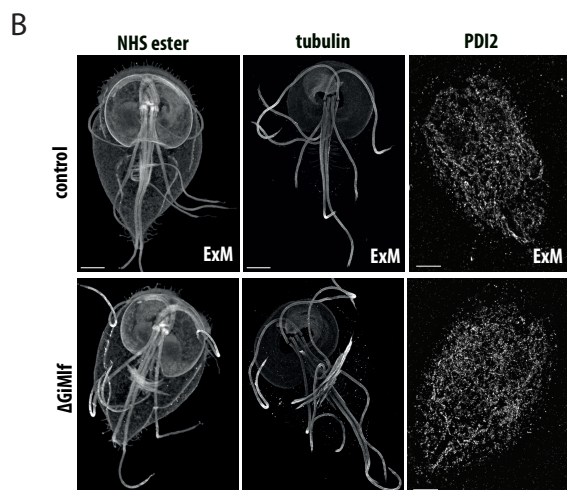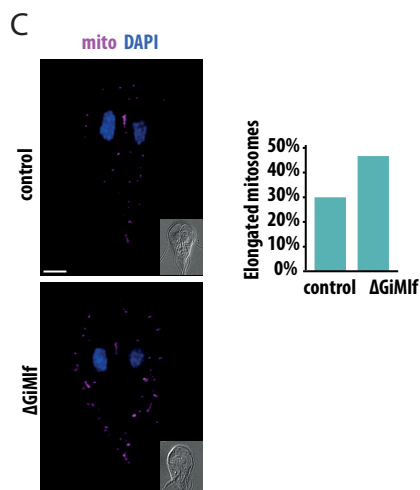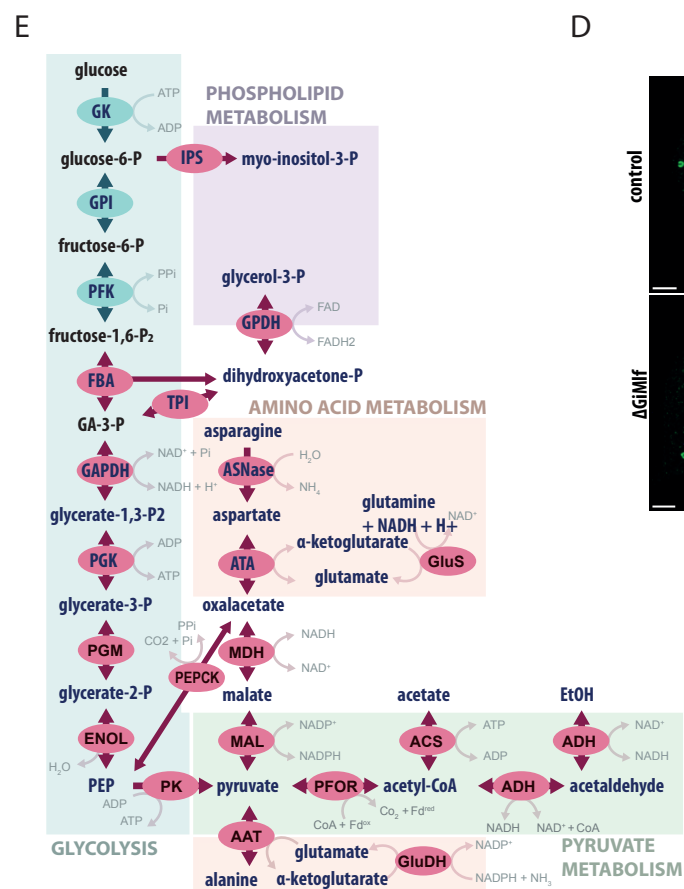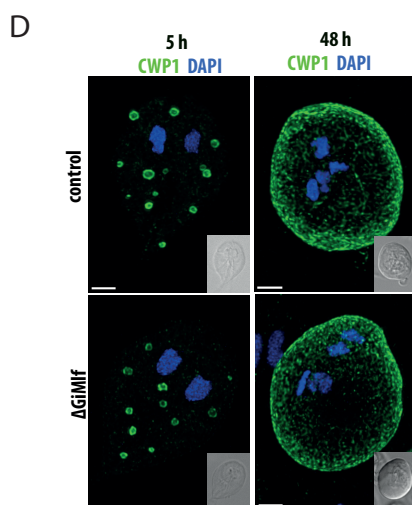

Supplement: S9 Fig — (A) Growth curves of ΔGiMlf and GiMlf-OE cell lines and their respective controls (n = 4). To establish whether the curves statistically differ, the CGGC permutation test (1000 permutations) was used. The growth of ΔGiMlf line was significantly impeded (P-value: 0.024), while there was no significant change in the growth of the GiMlf-OE line (P-value: 0.526) when compared to their respective control cell lines. (B) Comparison of the cell morphology of the ΔGiMlf and control cell line using expansion microscopy. Cells were stained with NHS ester, an anti-acetylated tubulin antibody, and an anti-PDI2 antibody. Scale bars: 10 μm. (C) Levels of the V5-tagged proteins in the control and ΔGiMlf cells in steady state (control) and after heat shock and 4h recovery. The proteins were detected by an anti-V5 antibody. TrxR was used as loading control. (D) Comparison of encysting cells (5 h and 48 h post induction) of ΔGiMlf and control cell lines using confocal fluorescence microscopy. The cells were stained with anti-CWP1 antibody (green). Nucleic DNA was stained with DAPI (blue). DIC images of corresponding cells are shown in corner of the merged images. Scale bars: 2 μm. (E) Schematic representation of Giardia energy metabolism with indicated downregulated enzymes (red). GK–glucokinase, GPI–glucose-6-phosphate isomerase, PFK–phosphofructokinase, FBA–fructose-bisphosphate aldolase, GA-3-P–glyceraldehyde-3-phosphate, TPI–triosephosphate isomerase, GAPDH–gleceraldehyd-3-phospate dehydrogenase, PGK–phosphoglycerate kinase, PGM—2,3-bisphosphoglycerate-independent phosphoglycerate mutase, ENOL–enolase, PEP–phosphoenolpyruvate, PK–pyruvate kinase, PEPCK–phosphoenolpyruvate carboxykinase (hypothetical protein GL50803_101278, homology inferred from HHpred prediction), PFOR–pyruvate-flavodoxin oxidoreductase, Fdox/red–oxidised/reduced ferredoxin, ADH–alcohol dehydrogenase, AAT–alanine aminotransferase, GluDH–glutamate dehydrogenase, ACS—Acetyl-CoA synthetase, MAL–malic enzyme, MDH–ma [file ppat.1012617.s009.pdf]
