# Supplementary material for: Mlf mediates proteotoxic response via formation of cellular foci for protein folding and degradation in Giardia
Source: PLoS Pathog. 2024 Oct 21;20(10):e1012617. doi: 10.1371/journal.ppat.1012617 (PMC11527388; doi:10.1371/journal.ppat.1012617)
Supplement: S10 Fig — (A) Fluorescence microscopy of the cell line overexpressing V5-tagged GiMlf. Top panel–Presence of large vesicular structures containing GiMlf in the vicinity of mitosomes. Stained with anti-V5 antibody (green) and anti-GL50803_9296 antibody (mitosomal marker, magenta). Nucleic DNA was stained with DAPI (blue). DIC image of corresponding cell is shown in corner of the merged image. Scale bar: 2 μm. Bottom panel–Expansion microscopy of the cell line overexpressing GiMlf-V5. Stained with anti-V5 antibody (green) and anti-PDI2 antibody (ER marker, magenta). Scale bar: 10 μm. (B) Transmission electron microscope image of the cell line overexpressing GiMlf-V5 and at low and high magnification, stained with an anti-V5 antibody. The blue arrow points to a peripheral vacuole. Scale bars: 200 nm. (DOCX) [file ppat.1012617.s010.docx]

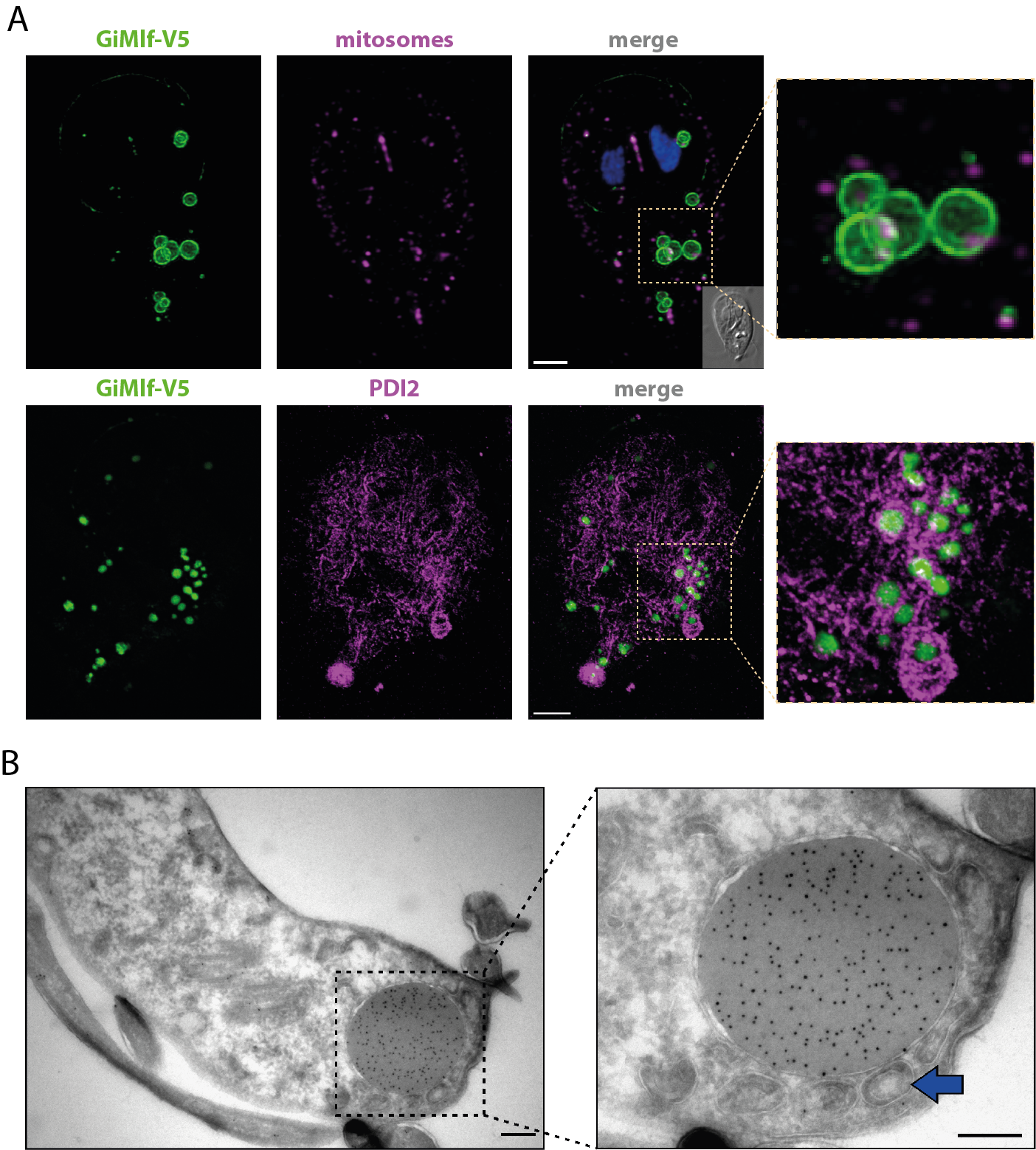


**Supplementary Figure 10.** **(A)** Fluorescence microscopy of the cell line overexpressing V5-tagged GiMlf. Top panel – Presence of large vesicular structures containing GiMlf in the vicinity of mitosomes. Stained with anti-V5 antibody (green) and anti-GL50803_9296 antibody (mitosomal marker, magenta). Nucleic DNA was stained with DAPI (blue). DIC image of corresponding cell is shown in corner of the merged image. Scale bar: 2 µm. Bottom panel – Expansion microscopy of the cell line overexpressing GiMlf-V5. Stained with anti-V5 antibody (green) and anti-PDI2 antibody (ER marker, magenta). Scale bar: 10 µm. **(B)** Transmission electron microscope image of the cell line overexpressing GiMlf-V5 and at low and high magnification, stained with an anti-V5 antibody. The blue arrow points to a peripheral vacuole. Scale bars: 200 nm.
