# Supplementary material for: Mlf mediates proteotoxic response via formation of cellular foci for protein folding and degradation in Giardia
Source: PLoS Pathog. 2024 Oct 21;20(10):e1012617. doi: 10.1371/journal.ppat.1012617 (PMC11527388; doi:10.1371/journal.ppat.1012617)
Supplement: Attachment — Submitted filename: Response to reviewers comments_MV.docx [file ppat.1012617.s015.docx]

**Response to reviewers comments**

**Part I - Summary**
Please use this section to discuss strengths/weaknesses of study, novelty/significance, general execution and scholarship.

Reviewer #1: The work is very interesting and original, and it contributes to the study in the field of parasitology, particularly regarding the parasite Giardia lamblia. The major strengths are the use of transgenic cells and various techniques to analyze the results. The weaknesses are particularly related to the conclusions drawn from the images showing the localization of the protein, which differ depending on the localization being analyzed. Therefore, I suggest showing photographs of a larger number of cells to trust these results. Additionally, some gels are of low quality, and the entire membrane should be shown. Finally, the study is novel, and if the suggested modifications can be made, it would have a significant impact in the field of giardiasis.

Reviewer #2: Vinopalová et al set out to elucidate the unifying function of Mlf that could explain its phenotypic
diversity. Comparative genomics and Alpha fold demonstrate that Mlf is conserved across the eukaryotic tree. In Giardia MLF was found to localize to basal bodies, the nuclear envelope, the periphery of the ventral disc, and in foci associated with mitosomes and ESVs. Interestingly heat shock upregulated expression of Mlf and the protein was observed to transition from a diffuse distribution to distinct foci. Further over expression of Get2 or Tom40 also caused upregulation of Mlf supporting the idea that Mlf has a role in proteostasis. KO of GiMlf resulted in changes in the levels of proteins regulating proteostasis and a change in the distribution of HSP 40 a conserved Mlf interactor. That GiMlf has a role in proteostasis is convincing. However, the manuscript needs revisions to correct statements about Mlf at MT nucleation zones. Rather it seems Mlf may have a role at the +end of MTs which has to do with growth and stability but not nucleation. Without nucleation we would expect to see a very perturbed MT cytoskeleton in the absence of Mlf, no such phenotype is observed in Mlf KOs.

Reviewer #3: This manuscript investigated the function of the Myeloid leukemia factor 1 in Giardia intestinalis (GiMlf1). The authors used several microscopy techniques (confocal, super resolution, and expansion) to localize giMlf1 more precisely to two areas in the cytosol: 1) microtubule nucleation zones where it interacts with HSP70; 2) in membraneless compartments next to mitosomes where it interacts with regulatory subunit 4 of the 26S proteasome (P26s4). Cellular stress induced by heat shock and by overexpression of organelle-specific membrane protein (Get2 of the ER or Tom40 of the mitosome) results in the relocalization of GiMlf1 from these sites. Interactome analysis provides evidence that GiMlf1 interacts with proteins involved in proteostasis or the regulation of protein quality control in the cell, and they showed colocalization of GiMlf1 with three of these proteins (Hsp40, Tom40, and P26s24). However, they did not see colocalization withYif1, despite it being one of the most highly enriched and most statistically significant proteins identified in the interactome. In general, their microscopy images showing protein colocalization are excellent although the colocalization of GiMlf with the basal bodies and ubiquitin are less convincing. Another lab published a paper (Wu et al., 2021) that showed the involvement of GiMlf1 in autophagy, encystation, and protein clearance in Giardia. Although this study also showed an involvement of GiMlf1 in protein clearance, they showed that a knockout of GiMlf1 likely affects encystation indirectly through disruption of general imbalance in proteostasis that induces a stress response. Furthermore, the authors did not identify proteins related to autophagy in their GiMlf1 interactome obtained from a biotin affinity purification assay with chemical crosslinking. Although the distribution of Mlf1 is found in most eukaryotes, a canonical function has not been determined. This study provides results indicating a role of the Mlf1 homolog in Giardia as a nucleator for protein assemblies to form membraneless compartments in the cytosol to regulate protein folding and degradation; this function may explain diverse Mlf1 phenotypes observed in other eukaryotes including humans.

Reviewer #4: The article titled "Mlf Mediates Proteotoxic Response via Formation of Cellular Foci for Protein Folding and Degradation in Giardia" authored by Vinopalová et al. presents a comprehensive study on the role of Myeloid leukemia factor 1 (Mlf1) homolog (GiMlf) in Giardia intestinalis. Mlf1 is known for its role in hematopoietic differentiation in humans and its involvement in a wide range of other cellular functions, including cell cycle regulation and ciliogenesis. This study aims to elucidate the cellular role of Mlf1 homologs in Giardia intestinalis, focusing on their involvement in protein folding and degradation during proteotoxic stress. The authors specifically present Giardia as a model organism suitable for elucidating the unifying function of Mlf that could make sense of its phenotypic diversity.

The authors show that GiMlf primarily localizes focally to two types of cytosolic structures: microtubule nucleation zones, where it interacts with Hsp40. Ubiquitin-associated, membrane-less compartments containing the 26S proteasome regulatory subunit 4, adjacent to mitosomes. They also provide evidence that GiMlf relocalizes or disperses in response to cellular stress, later forming enlarged foci during recovery. Recombinant GiMlf expressed in E. coli binds to signaling phospholipids on lipid strips, including phosphatidic acid (PA), phosphatidylinositol phosphates (PIPs), and cardiolipin (CL), suggesting a role in membrane recruitment.

They show a possible role of Mlf in proteostasis. Knockout of GiMlf leads to extensive proteomic changes indicative of compromised proteostasis. In addition, ΔGiMlf cells exhibit significant growth impairment, increased cyst production, and altered distribution of interacting proteins (Hsp40 and P26s4).

Taken together, the study underscores the critical role of Mlf proteins in maintaining cellular homeostasis under proteotoxic stress in Giardia by mediating the formation of apparently function-specific foci for protein folding and degradation. The findings provide an advancement in understanding Mlf's cellular functions in Giardia and potentially other eukaryotes. The methodological advance is notable: Utilization of CRISPR/Cas9 in Giardia demonstrated high efficiency in homologous recombination, facilitating the study of gene function without the need for antibiotic selection.

However, there are also some gaps as well as areas that merit much deeper exploration:
The study did not support previous findings on GiMlf's involvement in autophagy and its positive impact on encystation.
There is a lack of observation of membrane-bound GiMlf vesicles and autophagy-related factors in interaction assays.
The precise role of proteasomes in cyst production and viability remains unclear despite their colocalization with GiMlf in ESVs (encystation-specific vesicles).
While the findings are significant for Giardia, the extent to which they apply to other eukaryotes, including humans, remains to be further explored.
The interaction partners identified were not exhaustively validated functionally, leaving room for further investigation into their specific roles and impacts.
The authors performed a convincing phylogenetic analysis across the eukaryotic tree of life showing that the Mlf structure is highly conserved and not surprisingly found in very different roles in different organisms.
The narrative suggests that the goal of the overall investigation was at least in part to identify a unifying role for Mlfs using Giardia as a model (lines 60/61 and 87/88). However, this role has not been identified nor would it from the investigation of its function(s) in an additional, highly diverged eukaryote. Hence, in the light of the distinct possibility of diverse roles in Giardia itself I suggest that the text should be modified to tone down the rationale for this investigation.

**We would like to thank all reviewers for their careful, insightful and constructive reviews!**

**Part II – Major Issues: Key Experiments Required for Acceptance**
Please use this section to detail the key new experiments or modifications of existing experiments that should be absolutely required to validate study conclusions.

Generally, there should be no more than 3 such required experiments or major modifications for a "Major Revision" recommendation. If more than 3 experiments are necessary to validate the study conclusions, then you are encouraged to recommend "Reject".

**Reviewer #1:** Due to the fact that the localization of GiMlf changes according to the co-localizations that the authors want to highlight (it is clearly different in Fig 2A and Fig S2D), it would be advisable to include a photograph with a larger number of cells so that the localization of GiMlf can be more conclusively determined.

**We have included larger field image as Supplementary Fig. 3A. Also changed the exemplary image of the co-localization with the mitosomes in Fig.2A. These prolonged mitosomes are usually seen when the cell enters mitosis and it does not represent the dominant mitosomal morphology that is rather vesicular.**

Line 107: Fig S2D is not sufficient to conclude that the protein associates with PVs, as the resolution of the confocal microscopy is not definitive. Additionally, only one point of co-localization is shown. To assert this, more cells should be shown and perhaps another PV marker should be used, along with expansion microscopy. The DAPI is not visible in the photographs. The same applies to what is described as co-localization with ER. This could be complemented with the use of an anti-BIP antibody, for example.

**Yes, we agree that the evidence for the co-localization of GiMlf with the ER and PV is not sufficient. We thus removed this part from the manuscript.**

Supplementary Figure 2: The quality of the Western blot should be improved. Also, the entire membrane should be shown.

**The western blots were done again and changed in the figure. This time we used only C11 cell line, which was used for the subsequent analyses.**

Line 108: It is necessary to include more photographs of the different stages of encystation to observe how the localization of the protein changes.

**We think that this information is contained in the following Figure 3E.**

Moreover, in Figure 2D, a peripheral localization of GiMlf is observed, which could correspond to PVs or the plasma membrane. To clarify this, the overlap of that image with the DIC should be shown and an anti-PVs antibody should be used. Perhaps during encystation, the protein has a function within these organelles. On the other hand, this localization is not observed in Figure 3E, which shows the different stages of encystation.

**Unfortunately, we don’t have anti-PV antibody but the overlay with the DIC (see below) and also following data in the manuscript clearly demonstrate that this signal correspond to disc margin. Also, PVs are usually distributed evenly along the entire cell edge.**

**Also, it is definitely true that signal of GiMlf at the disc margin decreases significantly during encystation, but it can still be observed in selected layers from the microscopy (see below). At maximum projections, however, the signal becomes negligible. Please note, that the final stage of encystation shown at the bottom of Figure 3E has the disc already disassembled.**

Figure 2E should be shown in its original form, and then with the reconstructed cellular structures in color; otherwise, it is impossible to determine the protein's localization.

**We have added the projection of the tomography as a Supplementary Figure 2D.**

In Figure 3E, to show the re-localization of the protein, more cells should be shown again, as the pattern of GiMlf distribution, which is generally throughout the cell, is not very clear.

**The main purpose of the Fig. 3E is to demonstrate the co-localization of GiMlf with a proteasome component, and given the suggested role of proteasomes during the maturation of encystation vesicles, to follow their association during encystation. We believe that current data demonstrate this interaction well.**

Line 243: Again, this localization in what is called the disk margin is speculative and is not observed in most of the examples presented by the authors. It seems they select the right cell to draw conclusions. To generalize this, more cells should be shown, and co-localization with DIC should be performed.

**The disk margin is a term describing structural part of the giardia’s adhesive (ventral) disc and corresponds to the outer edge of the disc characterized by MT plus ends (E.g.. Nosala et al. 2018, Trends in Cell Biology). Please see the evidence provided above.**

Lines 258-263 are speculative and should be put in the discussion section.

**Yes, the text was removed.**

Figure 6A: The percentage of cysts is lower than generally reported in the literature, both using the two-step protocol and the Uppsala method (30%). The differences may be related to the method used to count the cysts. Generally, cysts cluster together, and when passed through the cytometer, they are discarded as doublets or triplets. I advise counting them using a classical method such as a Neubauer chamber. Additionally, to see if the cysts are viable, particularly in the case of the ΔGiMlf strain, it is advisable to leave them in water for 24 hours and then count them. This is because, under stress conditions, pseudo-cysts often form that do not mature into fully developed cysts.

**Thank you. We routinely use microcapillary flow cytometry (Guava) to quantify giardia populations (trophozoites and cysts) and do not observe clustering of cysts. This would show up on the cytometer anyway. We agree that publications report a higher encystation efficiency in vitro and we do not know the reason for this. It could possibly be the bile used for the encystation media. However, we used the same conditions for all cell lines tested, which makes the results comparable. We tested the viability of the cysts as suggested using incubation in water followed by Live/Dead staining, which showed that 84% of the ΔGiMlf cysts remained viable. This is less than the 94% viability established for the control strain, but the proportion of cysts formed by the ΔGiMlf strain is still more than double that of the control. Thus, we will definitely further investigate the encystation phenotype of the ΔGiMlf cells.**

**Reviewer #2:** Some specific experiments I would like to see would be 1.) to perform heat shock after treatment with cycloheximide to learn more about whether Mlf foci can re-distribute or if the foci which are often associated with ubiquitin are at the end of their life. 2.) Measure flagella length in control and Mlf KO cells particularly after heat shock. If a phenotype can be found this would support a role for Mlf in regulating MT length/dynamics (more below).

**Thanks for these suggestions. We performed heat shock after the treatment with cycloheximide and while the translation we effectively blocked, GiMlf still changed its localization, suggesting that the protein can re-distribute from the foci. However, we did not observe the background dispersed signal of GiMlf, which could correspond to the newly translated protein. We added this data into the text and as Fig.4E.**

It is said that Mlf associates with MT nucleation sites. This does not appear to be true. Mlf associates with basal bodies but away from where the MT nucleate. Also Mlf associates with the + ends of MTs in the ventral disc. It was incorrectly stated that MTs are nucleated at the disc margin but the Brown reference does not support this. Rather it states “59% of all microtubules in the disc are nucleated at dense bands 39% originate from the disc inner edge, and another 2% appear to have minus ends in the main body of the ventral disc”. Thus no MTs are thought to be nucleated at the disc margin where Mlf localizes. Those are the + or growing ends of MTs. Perhaps Mlf is part of the cap that stabilizes MTs or regulates their length. However not MT defects were observed in the Mlf KOs. Perhaps a MT defect could be uncovered by testing if the Mlf KO is hypersensitive to MT destabilizing drugs.

**We also measured the flagellar length in ΔGiMlf cells compared to control cells. Interestingly, we found that the flagella in ΔGiMlf cells were overall shorter by 6.2% with statistical significance (see below). However, when analyzed by flagella type, only the shortening of the caudal flagella was statistically significant. However, at the same time we observed that ΔGiMlf cells had an approximately 10% smaller volume. At this point, it was thus difficult to distinguish if the flagella length is a specifically related to flagellar stability or it just relates to the overall cell size reduction. Thus, we decided to follow the effect of GiMlf absence on flagella stability and dynamics in a more detailed future study.**

In several places it is stated that CRISPR KOs or editing can be performed without selectable markers, however selectable markers are used in both PT Guide and the Cas9 vector? I am not sure what the authors are trying to say with their various statements about not needing selectable markers.

**Yes, we agree that our statement is confusing. We edited the text so it is more clear. Briefly, what we meant is that for the in situ tagging of GiMlf we did not use puromycin selection marker in the recombination cassette, as was the case in the gene knock out design. Instead of flanking the PAC and the gRNA cassette with the homologous regions, here the template we used for integration of the tag was put in a different site in the pTG vector (see Fig. S2A). In this case, the PAC cassette was therefore not integrated into the genome. The cells were selected for both puromycin and G418 resistance after transfection to select cells that both expressed Cas9 (G418 vector) and possessed the vector with recombination cassette (puromycin). After confirming the integration of the recombination cassette into all alleles of the *gimlf* gene both on DNA level using PCR and protein level using western blot, the cell line was no longer selected for any marker. The cell line was further subcloned to ensure its homogeny. This way we also managed to gain population that altogether lost vector carrying the Cas9-G418 vector.**

**Reviewer #3:** No new experiments are required. Only clarifications or modifications of the presentation of existing data is requested that will be listed in the next part of this review.

**Reviewer #4:** Major issues that should be addressed by the authors:

Figure 2A shows an unusual pattern of mitosome morphology with stringy/elongated shapes, instead of the universally observed small spherical structures, like for example in Figure S7A – in addition, there is no data on validation of this antibody. See also below.

**We have changed the exemplary image of the co-localization with the mitosomes in Fig.2A. These prolonged mitosomes are usually seen when the cell enters mitosis and it does not represent the dominant mitosomal morphology that is indeed vesicular. This antibody has been established in our 2015 paper Martincova et al. in MCB and used in several papers on mitosomal biology.**

Figure 2B/C: expansion microscopy should be accompanied by regular fixation and labeling of cells for comparison which would help to determine whether artefacts are produced. Also, full images of the entire cells including frames for regions of interest should be shown for orientation and to help gauge the reproducibility of the subcellular distribution.

**We use expansion microscopy only for the dense regions of the cell which cannot be seen by regular fixation. It is also the case of GiMlf which can be clearly observed at this region only after the denaturation and expansion step. Please see below the whole cell images with ROIs and another expanded cells showing analogous labelling. We have added the whole cell images to the Supplementary Fig. 3.**

Figure 2E: An overview picture with and without reconstructions should be provided. The question of association of gold particles to specific structures cannot be definitively answered from just one subsection of the cell. Additional imaging data with multiple replicates and transparency about the workflow for segmenting and identifying structures is required to support the interpretation in the text.

**We have added the projection of the tomography without segmentation as a Supplementary Fig. 4. Of course, we used two control labelings. First, only secondary antibody with the conjugated gold particles was used on the experimental strain, and this information is in the Method section. The immunolabeling by primary anti-V5 antibody was also tested on giardia cells without the in situ tagged GiMlf using the same anti-V5 antibody. We added this information into the Method section as well. Below are other examples of immunogold labeling showing the specificity of the procedure.**

Figure 2I: the cloudy background on the delta NT probed lipid membrane on the right is very unfortunate. Also, the interpretation should be that the deletion of the NT disordered region simply partially attenuated the binding strength but not the specificity. Line 891: what was the control?

**It is usual that lipid strips show higher background then regular western blots due to less stringent washing conditions. So, in our experience it is still solid piece of data. Considering the interpretation of the data in Fig. 2I we wrote: “**The interaction with PIPs appears to be tied to its N-terminal disordered region, as evidenced by the significantly reduced binding properties of the N-terminally truncated variant lacking the first 53 amino acids (GiMlf-ΔNT) (Fig. 2I).” **In this experiment, the PA and cardiolipin binding remained unaffected, and only binding to PIPs was reduced, so we think is it what the above sentence is saying.**

**The sentence about the control actually belongs Fig S2G now Fig. S3C and remained there after we changed the figure arrangements. As a negative control we probed the membrane with a His-tag peptide to exclude the nonspecific binging od the recombinant His-tagged protein to the lipid strip. This information was added into the legend of Fig. S3C.**

Most importantly, there is no mention of the CT conferring strict specificity to cardiolipin as shown in Fig S2G! This is in fact what one would expect after deletion of a binding domain as opposed to the mere attenuation elicited after deletion of the NT domain. Cardiolipin is an essential component of mitochondrial membranes which could explain localization of GiMlf to mitosome organelles although assignment of the lipid to mitosomes has yet to be shown.

**Thank you for this insightful observation. We did not address this point because binding to cardiolipin was not reproducibly abolished when other versions of the protein truncated from the C-terminus were used (see below). Thus, at this point we are unsure of the actual results, whether they reflect true binding to cardiolipin or just misfolding of the protein, etc. We will investigate this further, but we are removing the C-terminal truncation data from the paper to reduce confusion.**

Lines 135ff : Evidently, a rationale and research question was developed leading to testing the influence of the N-terminal part of Mlf in lipid binding. However, the authors wrap the question (or rather statement) and the answer into one sentence. This also glosses over the fact that deletion of the NT domain yields the same albeit a significantly weakened pattern on the lipid strips in addition to disregarding the key role of the CT for binding to cardiolipin.

**See above.**

Figure 3E: none of the ROIs on the right are in fact enlargements of the merged images – they appear to show a different plane. This begs the question whether the images shown here are indeed “maximum intensity projections” (line 478). This should be fixed. Please note that the same discrepancy is seen in Figure 5A.

**Thanks. In fact, in the legend to Fig. 3E it is stated that: „The enlarged images show a single layer of the image stack” but the legend for Fig. 5A misses this information. We prefer to show single layers for the ROI to demonstrate the co-localization (please see below). We changed the text so there is no confusion.**

Figure 3E

Figure 5A

Figure 4A: The increase in GiMlf is appreciated, but there is no measure in this figure and 4B of how much overexpression was achieved and the text includes neither this information nor the method with which it was achieved. Both should be incorporated in the main text and 4A and B should be expanded to show baselines of all proteins before and after overexpression.

**This experiment was done by simply transforming cells with plasmids encoding these membrane proteins under relatively strong promotors. This information was added into the Methods section. Moreover, we don’t have an anti-Get2 antibody, which would allow to quantify the level of overexpression. We did this experiment just to demonstrate that GiMlf changes its localization if a protein is overexpressed in the cell over its natural level. Here, we repeated the western blot and replaced Figure 4A, adding the calculated upregulation to the text (the upregulations were statistically significant using a t-test with unequal variance, one-tailed). Mlf was upregulated 2-fold in the Get2 strain and 2.1-fold in the Tom40 strain. Tom40 was upregulated 4.1-fold in the Tom40 strain.**

The quantification of the signals on the Western blot is not valid in my opinion as many of the bands are not in the (relatively narrow) linear range anymore and plateaued. This analysis can only be done with dilution series and measurements in the linear range.

**The signals were OK for quantifications. Please see below density readings when analyzing the western blot(s).**

Lines 211/212: First off, a heat shock provoking a heat shock response is kind of a given. .But this also assumes that a mere 6°C difference actually results in massive protein unfolding. Why not make the experiment more specific by using for example DTT to induce a UPR (see e.g. Ravi, Kumar A, Bhattacharyya S, Singh J. 2023 EMBO J.)?

**Yes, we agree that the heat shock response may seem like an 'old-fashioned' way to stress cells. However, it's important to consider that it is one of the most common natural stress factors, and even a slight temperature increase can cause dramatic changes in transcription and protein folding, playing a central role in cellular homeostasis (e.g., Richter et al., 2010, Mol. Cell). On the other hand, exposing cells to a strong reductive agent like DTT, which they dont encounter in their natural environment, seems too harsh. Moreover, the unfolded protein response (UPR) has not been demonstrated in giardia and even suggested to be missing (Spycher et al, 2013). We also try to avoid the use of DTT in anaerobes like *Giardia*, which rely heavily on the activity of Fe-S proteins, DTT causes further complications by removing Fe-S clusters from these proteins.**

Figure 4C: Documentation of this experiment is insufficient as the bands shown on the Western blot are mostly not in the linear range for densitometric analysis. This experiment should be repeated and expanded to include dilution series and measurements in the linear range for all markers together with the corresponding statistics. Alternatively, a qualitative assessment might be sufficient.

**Please see our comment above. The densitometric readings are fully quantifiable. Also the saturation would be simply identified by the detector. Also the variations across replicates do not suggest that the readings are plateaued.**

Line 276: What are elongated mitosomes? So far, only spherical organelles have been documented in Giardia. The sentence suggests that there is a population of elongated organelles at steady-state in trophozoites. However, EM studies of the central mitsosome complex has shown consistently that this consists of a grape-like arrangement of individual small spherical organelles embedded withing the basal body complex, whilst small spherical peripheral organelles do not normally aggregate or assume an elongated shape. In this context the bar graph figure in 6C is not quite understandable.
The observed phenotype is striking, however, and indicates a different type of organization of peripheral mitosomes which appear to form linear arrays.

**In addition to concerted division during mitosis, we sometime observe individual elongated mitosomes or mitosomes arranged into the arrays. However, we agree that this require specialized study and have therefore removed this data from the paper for a future study as it introduces unnecessary side line.**

Line 280: Encystation does not appear to be correlated with stress response - at least hallmarks of a stress response due to media changes or protein overload in trophozoites stimulated to encyst are not detected. I believe this has been clarified in Morf et al. 2010. This is actually in line with the ability of trophozoites to persist for weeks in all areas of the intestine using immune-evasion strategies such as antigenic variation. In addition, encystation is not confined to the distal parts of the small intestine where we find a paucity of lipids as the Müller group showed in mice.
Subtle increases in pH such as from the proximal to the distal small intestine and zones of cholesterol depletion are not stressors per se but may trigger signal transduction and elicit a transcriptional response. Hence, there is no evidence for stress as an explanation. Why not just state that this unexpected result requires further research?

**Yes, we agree. The sentence was purely there to hypothesize the cause of the phenotype. We have changed the sentence accordingly.**

Figure 6: Mislocalization is an over-interpretation and implies a loss of function (since Hsp40 is not at its normal site of action anymore). It’s conceivable that Hsp40 relocalizes completely within the range of its intended function to compensate for the absence of GiMlf.

**Yes, it is true, thanks. We have changed that.**

**Part III – Minor Issues: Editorial and Data Presentation Modifications**
Please use this section for editorial suggestions as well as relatively minor modifications of existing data that would enhance clarity.

**Reviewer #1**: Line 31: An introductory phrase related to Mlf1 should be added.

**We modified the introductory part a bit.**
Line 47: The citation of Adam is a review. The original paper should be cited.

**We have added two more reference, although both of which are reviews, but more on cell biology. Of course, the absence of the Golgi complex and peroxisomes will stand until experimental data show otherwise.**

Line 174: It should say Fig S3D instead of Fig S2D.

**Corrected**

In Fig S7A, the letters in figure A are not readable.

**We don’t know what happened here, it perhaps happened during conversion on the journal website**

**Reviewer #2:** Line 93: insertion of the (BAP) tag into all four alleles of the gimlf gene without requiring antibiotic selection. Figure S2A indicates that pTguide contains a PAC cassette. So this statement is confusing to me. Do you mean that after editing and selection that integration into the genome allowed maintenance of the strain without antibiotic selection? Or I notice that the schematic shows the Mlf 5’ and 3’ homology regions are only for including the BAP tag, so is it the case that the PAC cassette and gRNA cassette do not get integrated into the genome.

**Yes, that is correct, no selection after integration was necessary, and the PAC and gRNA cassettes do not integrate into the genome. We have changed the wording in the manuscript, so it is more clear.**

Not a criticism or issue but I am wondering what advantage the BAP tag has over a standard pulldown or TurboID approach. It seems a more complicated approach. It worked so no issue to address, I would just like to understand why one would select this approach?

**It is just a well-established protocol in our lab that gives us minimal background binding. Of course, we would prefer to use TurboID or other versions of promiscuous BirA but they don’t seem to work in Giardia, at least in our hands.**

Figure S2B It would be helpful to add a schematic of where the PCR primers are positioned relative to the tag and chromosomal DNA, perhaps as part of S2A. The results for Mlf in the genome is confusing because this experiment was to edit Mlf with the BAP tag so we expect it to be present. I notice a slight shift so perhaps the point is to show that the tag is present in C11, D6, and E7. Also what was used as the NC?

**Correct, we added the primer positions into the schema, part of Fig. 2SB**

Figure 2. We were not told how many cells were examined for each localization and if the experiment was repeated multiple times to ensure reproducibility. Is Mlf associated with a subset of mitosomes in every cell or is the association rare. Quantification is important to make this clear. Also if infrequently observed it could just indicate transient interaction. Same issue for 2B, 2C, 2D.

**These are very stable interactions apart from the stress conditions. In vast majority of cells, one can find GiMlf associated with the mitosomes, but not vice versa – there are mitosomes that do not have GiMlf signal. And the same with the cytoskeleton and ESVs. There will always be a signal of Mlf protein associated with the cytoskeleton and ESVs. We have added this information in the text, so it is clear. So, concerning the numbers, it is hundreds of cells throughout the study. We have added the larger field image of more cells in a Supplementary Figure 3A, which should demonstrate the situation. When the association of mitosomes with GiMlf is counted it is about 80% of mitosomes with clear GiMlf signal (here done on 25 cells).**

Line 102: The dense band statement is likely wrong. The dense band shown by Hagen should be in the bare area of the ventral disc. Brown 2016 shows there are 6 dense bands and they are very close to the basal bodies. The image is zoomed in so my orientation could be off. Localization to the disc margin and basal bodies is convincing, but more needs to be done to verify dense band localization. Since there are no known markers to be used for co-localization I don’t have any specific recommendation on how to confirm dense band localization. Perhaps the authors can provide additional images that can be compared with the Brown and Hagen results.

**We think that the area labeled in the middle image (dense bands, in Brown et al. 2016) corresponds to the immunogold labeling (red arrow) and the green signal in the IFA (red arrow)**

Line 104 “margin of the adhesive disc (Fig. 2C,D), where approximately 39% of the microtubules nucleate (Brown et al., 2016)” The Brown 2016 reference is not listed in the bibliography. More importantly, this statement is an incorrect understanding of the literature. The paper indicates that 59% of all microtubules in the disc are nucleated at dense bands 39% originate from the disc inner edge, and another 2% appear to have minus ends in the main body of the ventral disc. So no MTs are reported to be nucleated at the disc margin.

**Yes, we corrected it.**

Line 174 “Hsp40 was either present at the axonemes” After looking at Figure 3C it would be more accurate to say at the basal bodies since HSP40 doesn’t mark any other part of the axoneme in WT cells.

**The text goes: ….“The Hsp40 was either present at the axonemes (52% of observed cells, n=50) (Fig. S4D) or displayed ubiquitous cytoplasmic localization, appearing also at the flagella (48% of observed cells) and in 40% of cells it was additionally present at the disc margin, where it colocalized with GiMlf (Fig. 3B). ……” In the main figure we show only the sites, where the proteins co-localize. Otherwise Hsp40 can be found in the axonemes, which is shown in the Supplementary Figure 4D.**

Line 178 “the dense band of the adhesive disc (Fig. 3C)” Figure 2C claims to show the dense bands of the disc which I disagreed with above. Figure 3C only shows basal body and disc margin localization? Perhaps this text was meant to be above when discussing Fig 2. However, the authors should drop this statement or provide better images. I think there is good support for Mlf at +ends of MTs but not at -end nucleation sites. In fact, I am unaware of any other MT binding protein associating with both + and – ends.

**Please see our reasoning for this statement above.**

Line 188 it would be more accurate to say remodeling rather than redesign

**Done**

Figure 4D is very interesting and I appreciate the quantification. One experiment that could help us understand the relationship between stress and puncta vs ubiquitious distribution would be to treat the cells with cycloheximide before the heat shock. This would block new protein production and allow us to determine if the puncta disperse. Perhaps the puncta are the end of the story and the protein always starts disbursed and then aggregates.

**Please see our response to this point in the Major points section.**

Fig 5A. How many cells were examined and how many independent experiments were performed? Other figures indicate n=50, this is on the lower end for quantification of fixed cells with standard imaging approaches. Our experience has been that values stabilize after about 200 cells. I suspect if another 50 cells are quantified the presented values would be different.

**Yes, we usually analyze 50 cells. But in this case, we haven’t done any statistical analysis to support this observation. However, the recovery after the stress reproducibly leads to the increase in bodies containing GiMlf and Ub.**

Line 252 here a comma is used instead of a period and in other places a comma is used to indicate thousands. Edit this instance for consistency.

**Done**

Line 258 “indicates that no GiMlf-positive, membrane-bounded vesicular structures of appropriate size could be observed by electron microscopy after immunogold labeling” I don’t think the authors can be confident about this statement. Consider that there are only a handful of foci per cell and data is presented for phospholipid binding which would indicate association with membranes. How many sections were analyzed? Was the EM performed on heat shock recovered cells that were shown to have a large increase in Mlf foci?

**We removed the entire paragraph**

Figure 6B. The caudal flagella appear longer in control cells after heat shock recovery. This should be quantified by measuring flagella length. If true it would help point towards a function at flagella tips (+ ends of MTs).

**Yes, as state above, we found caudal flagella to be longer with statistical significance but we need to investigate this phenotype specifically in relation to the cell size, which also appears to be affected.**

Line 355 I don’t believe basal body localization should be equated with the MT nucleation zone. It seems that Mlf is not at the business end. Also there is discussion of Mlf’s role in cilliogenesis, but GiMlf was never demonstrated to be in the axonemes, we only saw that HSP40 is at the + ends of MTs after heat shock in the Mlf mutant. Perhaps Mlf has an indirect role by regulating HSP40 which does have a role in cilia motility (Zhu 2019 MBOC).

**Thanks, we have changed the sentence so it does mention only the actual cytoskeletal structures in giardia and not the nucleation zones.**

Line 378 indicates that KOs are made without selectable markers but selectable markers are used. This statement makes no sense to me.

**The sentence was changed to make sense. We meant the selectable marker in the recombination template.**

Line 425 punctuation

**Done**

Antibodies listed in the methods: The specific antibody name should be provided to avoid ambiguity- For example Cell Signaling has multiple anti-ubiquitin antibodies and Sigma has multiple anti-tubulin antibodies.

**Done**

**Reviewer #3:** 1. Fig. 1 and Fig. S1: it is strange that GiMlf1 groups with the Euglenozoa in the phylogenetic trees. The authors should include a comment about this. Also, do Trichomonads and Entamoeba have MLF1 homologs?

**In this phylogenetic analysis, giardia GiMlf1 cluster with other metamonad sequences (Spironucleus, Trepomonas). Together metamonads appear as a sister branch to Euglenozoa and Heterolobosea. This is in agreement with metamonads being a part of Excavata together with Euglenozoa and Heterolobosea. Of course, the tree is based on a single protein sequence and does not contain the information to resolve such deep branches. While trichomonads have multiple Mlf homologues, no sequence has been identified in Entamoeba. We have added the information on the presence of Mlf homologues in other metamomad sequences into Result section.**

2. Fig. 2: the colocalization of GiMlf1with the basal bodies is not very clear even in the expansion microscopy images. Is there a reason why the authors did not attempt to colocalize the GiMlf with centrin since a centrin antibody (Millipore 04-1624) that works in Giardia is available?

**Yes, thanks. We agree that centrin would be a great marker for the basal bodies. However, we did not have access to the centrin antibody in our lab, and the two-month revision window was insufficient to acquire and optimize the use of a new antibody also considering other experiments. We also think that the immunogold labelling also provide additional information along this line (we added tomography data without segmentation). But, we will follow several aspects of GiMlf in giardia biology including the precise localization of the protein signal at the basal body.**

3. Lines 106-107: "a minor fraction of the protein was associated with other membrane-bound organelles including the nuclear envelope (Fig. 2C)." I am not sure if the association of GiMlf1 around the nuclear envelope should be considered “minor”. This adverb should be removed.

**Done**

4. Lines 216-217: "increase in GiMlf protein levels was more gradual, reaching a peak at 4 hpi., and could only be detected after 30 minutes of the recovery period". This sentence needs to be corrected as Fig. 4C shows a band for GiMlf even at 0 h.

**Yes, the sentence was confusing, we meant that “the increase” could be detected…..We changed the sentence.**

5. Lines 216-264: "It is plausible that these foci are in fact built by the oligomerized GiMlf subunits as high molecular weight species corresponding to GiMlf octamers, as observed by size exclusion chromatography of purified recombinant GiMlf(ΔCT) (Fig. S5)". How did they get an octamer from the size exclusion that shows peaks at 36 kDa and 163 kDa for GiMlf(ΔCT). The form at 163 kDa is closer to a tetramer.

**We have used shorter ΔCT version of the protein. The size of the GiMlf(ΔCT)** **monomer is 22 kDa. We reasoned that 36 kDa species might be a dimer, and 163 kDa peak an octamer.**

6. Lines 284-285: "The amounts of the V5-tagged proteins were comparable to those in control cells, but their cellular distribution was significantly altered in the ΔGiMlf cell line." Where is the western blot that show the levels of the V5-tagged proteins in ΔGiMlf is the same as those in the control cells?

**We added the western blot into a Supplementary Figure 6.**

7. Line 413. The catalog number of the bovine bile used for encystation should be given as different types of bovine bile are available from Sigma and they have differences in their ability to induce Giardia encystation.

**Added**

8. Line 413: the efficiency of encystation was determined by flow cytometry. These % efficiencies should be given.

**These numbers are shown in the Results section**

9. Line 582: "Giardia trophozoites were grown in 10ml plastic tubes with flat bottom (Thermo Scientific) to full confluence". Why were “flat bottom” tubes mentioned? Is this important for the thermal stress experiment? If so, they should say why and give catalog numbers for these tubes. A better way of phrasing this would be to say "were grown in flat-bottomed 10 mL plastic tubes".

**No, there is no specific need for these tubes. We removed the sentence.**

10. Growth curves were plotted with cell counts from using a using Guava easyCyte 8HT (Luminex). Were the cells stained with a viability dye or other dyes for counting?

**No viability dye was used.**

11. More information about the source of some of the antibodies is needed. Where or how were the antibodies for PDI2, enolase, GiMlF1, Bip, and thioredoxin reductase obtained? Were they made in the authors’ lab, obtained from another lab, or commissioned as a custom antibody from a company? What were the antigens used: – peptide or recombinant protein?

**The information was added into Methods section.**

12. Line 891: CT – control – what was the control? There is no sample labeled as CT in Fig. 2I.

**This was a typo, it was corrected.**

13. Fig. 4C: why did the authors choose to use thioredoxin reductase as a loading control in the western blot? Did they try to use common housekeeping proteins such as actin as a loading control?

**The choice was primarily practical. After testing all available antibodies, we found that thioredoxin remained stable during thermal stress and provided more reproducible results compared to other proteins, such as tubulin and enolase**

14. Line 1055: 10xx diluted. Typo needs to be fixed to read "ten-fold"

**Corrected**

15. Supplementary Table 2 Table of proteins with significant changes in expression in the GiMlf knock-out cell line compared to the control cell line Cas9-pTGUIDEempty. What is the “NaN” entry in the “fold-change” column? Are these proteins identified in the KO cell lines that are NOT present in the control cell lines? Therefore, these are proteins unique to the KO cell lines.

**These proteins were initially identified as unique to the KO cell line or to the control cell line. However, many of them belong to large protein families, such as variant surface proteins, whose expression can fluctuate during cultivation. Additionally, several of these proteins were detected only at the limit of the mass spectrometer’s sensitivity or were not consistently identified across all replicates. Based on our extensive experience with similar pulldown experiments, we do not consider these proteins to be truly unique.**

**Reviewer #4**: Minor Issues:

Line 128: The title should be revised to fit with the text: e.g. Recombinant Mlf binds structural and signaling membrane lipids in vitro.

**Yes, we changed the text accordingly.**

Line 129f: Given the data presented up to this point, the claim is rather surprising. The authors should explain.

**OK, it may sound confusing. We slightly edited the sentence.**

Figure S6F is mislabeled

**Done**

Lines 133/34: The sentence is unclear and should be revised.

**Done**

Lines 366f: That's an appealing idea, but not supported experimentally in this work. It has been shown that stress response and encystation are not coupled at least in the sense that the former does not induce encystation and the latter does not elicit a stress response. The informative experiment would be to test whether the rate of spontaneous encystation is increased in KO cells. This would require complementing ectopically with a dd-tagged Mlf gene and stabilization with Shield/Rapamycin to reverse the effect.

**Yes, we understand, we edited this part of the discussion.**

Lines 358ff: The authors state “Considering our data alongside these independent findings, it seems plausible that the unifying function of Mlf proteins is to mediate the formation of concentrated function …”. They set out to identify a unifying function in a poorly characterized model system. This conclusion is premature and excludes many possible other scenarios.

**Yes, we removed this generalizing sentence.**

PLOS authors have the option to publish the peer review history of their article ([what does this mean?](https://journals.plos.org/plospathogens/s/editorial-and-peer-review-process#loc-peer-review-history)). If published, this will include your full peer review and any attached files.


If you choose “no”, your identity will remain anonymous but your review may still be made public.


**Do you want your identity to be public for this peer review?** For information about this choice, including consent withdrawal, please see our [Privacy Policy](https://www.plos.org/privacy-policy).

Reviewer #1: No

Reviewer #2: No

Reviewer #3: No

Reviewer #4: No
